# Does 3D volumetric carotid plaque imaging by cardiovascular MRI solve the mystery of the 'percent stenosis paradox'?

**DOI:** 10.1186/1532-429X-11-S1-P22

**Published:** 2009-01-28

**Authors:** Robert WW Biederman, Ronald B Williams, Saundra B Grant, Geetha Rayarao, June A Yamrozik, Vikas K Rathi, Diane A Vido, David R Neff, Mark Doyle

**Affiliations:** 1 Allegheny General Hospital, The Gerald McGinnis Cardiovascular Institute, Pittsburgh, PA USA; 2 Merck/Schering-Plough Pharmaceuticals, Inc., North Wales, PA USA

**Keywords:** Carotid Plaque, Plaque Composition, Carotid Artery Disease, Lipid Pool, Fibrous Plaque

## Introduction

Aggressive pharmacologic, dietary and exercise strategies have been employed in recent years to dramatically lower incidence of CAD as evidenced by striking reductions in MI and CVA and other cardiovascular complications. Yet, when examined at the pathologic source, namely the arterial lumen, little if any changes are seen. Typically, luminal % stenosis changes are nominal after aggressive statin intervention, often <5%, yet translate clinically into substantial reductions in morbidity and mortality. The explanation for this dichotomy remains unclear. Recently, we and others have noted that morphologic characteristics of plaque composition help to explain the apparent paradox of improved clinical events despite no or minimal reduction in % stenosis with statins. In that CMR can distinguish underlying features that determine plaque 'vulnerability', we sought to define the role of composition vs. %stenosis.

## Hypothesis

We hypothesize that in statin-naive pts with high-grade carotid artery stenosis, there will be a high degree of correlation in the relationship between the 'unstable' lipid pool and 'stable' fibrous plaque by 3D CMR, yet may be independent of 2D luminal %stenosis.

## Methods

Representing 530-two mm contiguous CMR (1.5 T GE, EXCITE, Milwaukee, WI) *in vivo* slices of advanced (> 50%; mean 61 ± 24% stenosis) carotid artery disease, 26 complete bilateral human (age: 66 ± 14 yrs) plaques were analyzed for 3D volumetric extent of vascular wall: lipid pool, fibrous cap, matrix and minima/maxima of each. All were related to fasting lipid levels relative to %stenosis via QPlaque (Medis, The Netherlands). The plaque morphology was determined by T1, T2/PD CMR, chiefly reliant on the former.

## Results

In all, 25/26 *in vivo* plaques were successfully imaged and quantified volumetrically. Mean resolution: 1 × 1 × 2 mm. The mg/dL range of LDL-C was 63–186, HDL-C: 28–59 and TG: 81–213. Lipid pool represented 15 ± 4%, while fibrous plaque represented 5 ± 15% of total vessel wall. Total Cholesterol (Chol_T_) and LDL-C were inversely related to minimum vessel wall thickness (r = -0.5 and -0.6, respectively, p < 0.05 for both) while only Chol_T_ was related to fibrous cap (r = 0.6, p < 0.01). The Chol_T_/LDL-C ratio was highly related to minimum fibrous plaque thickness (r = 0.8, p < 0.001). The 3D lipid pool was the only fraction highly correlated (>0.6) with triglycerides (r = 0.6, p < 0.01). A linear regression relating fibrous cap: vessel wall ratio to non-HDL cholesterol and Chol_T_ was highly correlated (r = 0.6, 0.7, respectively, p < 0.01 for both) but was independent of *in vivo %* stenosis (r = 0.1). Relating % luminal stenosis to *any* lipid fraction, subfraction or to any vessel wall component or its ratio revealed no statistically significant relationship.

## Conclusion

Percent stenosis provides relatively little information about vulnerability of *de novo*, statin-naive carotid plaques in high-grade carotid artery disease. As most current imaging based clinical studies (IVUS, MRA, CTA and contrast angiography) monotonously concentrate on plaque stenosis for identification of timing for intervention, a more appropriate focus on underlying plaque composition may provide a more robust quantifiable volumetric metric potentially more indicative of the underlying pathology and vulnerability by high-resolution 3D CMR.

